# Anti-EGFR function of EFEMP1 in glioma cells and patient prognosis

**DOI:** 10.18632/oncoscience.24

**Published:** 2014-03-25

**Authors:** Yuanjie Hu, Hengjun Gao, Christopher Vo, Chao Ke, Francine Pan, Liping Yu, Eric Siegel, Kenneth R. Hess, Mark E. Linskey, Yi-Hong Zhou

**Affiliations:** ^1^ Department of Biological Chemistry, University of California Irvine, Irvine, CA, USA; ^2^ Institute of Digestive Disease, Tongji University, Shanghai, China; ^3^ National Engineering Center for Biochip at Shanghai, Shanghai, China; ^4^ Neurological Surgery, University of California Irvine, Irvine, CA, USA; ^5^ State Key Laboratory of Oncology in South China and Collaborative Innovation Center for Cancer Medicine, Sun Yat-sen University Cancer Center, Guangzhou, China; ^6^ Ziren Research LLC, Irvine, CA, USA; ^7^ Department of Biostatistics, University of Arkansas for Medical Sciences, Little Rock, AR, USA; ^8^ Department of Biostatistics, The University of Texas MD Anderson Cancer Center, Houston, TX, USA

**Keywords:** EFEMP1, EGFR, glioma, prognosis, tumorigenicity

## Abstract

EGFR is one of the key oncogenes subjected to targeted therapy for several cancers, as it is known to be amplified and/or mutated in up to 40% of malignant gliomas. EFEMP1, a fibulin-like extracellular protein, exerts both tumor suppressive and oncogenic effects in various cancers and glioma cell models. Although EFEMP1's anti-cancer activity has most commonly been attributed to its anti-angiogenic effects, we showed for gliomas that EFEMP1's binding to EGFR accounts for its suppression of the intracranial tumorigenicity of glioma cells expressing high levels of EGFR. In gliomas where *EFEMP1* expression, and thus the anti-EGFR effect of EFEMP1, was suppressed, heightened levels of *EGFR* expression were associated with unfavorable patient outcomes in prognostic models. Results from the current study clearly demonstrate the impact that the anti-EGFR function of EFEMP1 has on the expression of *EGFR* and patient prognosis. A glioma prognostic model also suggests EFEMP1's context-dependent oncogenic function in gliomas expressing low levels of EGFR. Hence the level of *EFEMP1* expression may have a predictive value for choosing patients for anti-EGFR therapy.

## INTRODUCTION

EFEMP1 is a member of the fibulin family of secreted glycoproteins containing a series of epidermal growth factor (EGF)-like modules, followed by a carboxy-terminal fibulin-type module. Fibulins are hypothesized to function as intramolecular bridges within the extracellular matrix to form supra-molecular structures, and as mediators for cellular processes and tissue remodeling, and hence can be involved in cancer [1-3]. Studies of Efemp1 knock-out mice have demonstrated the role of Efemp1 in maintaining tissue integrity, stimulating the expression of Timp1 and Timp3, and inhibiting the expression and activity of matrix metalloproteinases Mmp2, and Mmp9 [4, 5]. In humans, EFEMP1 was initially identified as a senescence protein [6, 7]. Malfunction or deregulated expression of EFEMP1 has been implicated in retinal dystrophy, Werner syndrome, adult height, and cancer (see review in [8].

The anti-cancer properties of EFEMP1 were initially thought to be derived from direct targeting of endothelial cell proliferation to suppress angiogenesis in cancer [9]. The tumor suppressive role of EFEMP1 has been confirmed in cancers from several organs, including brain, breast, colon, lung, liver, nasopharynx, and prostate [10-16]. Anti-cancer correlations with improved patient prognosis have been based on findings of hypermethylation of the *EFEMP1* gene [10-16], down-regulated *EFEMP1* expression in cancer specimens and derived cancer cell line [10-12, 15-16], and positive correlations of *EFEMP1* expression with suppressed lymph-node metastasis [14, 16] and overall survival [11-17]. Anti-cancer effects have also been confirmed *in vitro* through characterization of EFEMP1 function in cell lines derived from human lung cancers [10, 18], nasopharyngeal carcinomas [14], and malignant gliomas [17]. In addition to observation of angiostatic effects, additional mechanisms underlying EFEMP1's tumor suppression function that have since come to light include attenuation of EGFR/AKT-mediated growth signaling activities [14, 17] and reduction of MMP-induced cancer cell invasion [18].

A potential oncogenic role of EFEMP1 was identified in cervical carcinoma [19] and pleural mesothelioma [20], and has been found in a human pancreatic carcinoma-derived cell line [21], a chemically induced rat glioma cell line, and in human glioma-derived stem-like glioma cells [22, 23]. The above reported findings showed dual functions of EFEMP1 in cancer as being both tumor suppressive and tumor promoting. Within specific cancer cell contexts, EFEMP1 may promote [24] or suppress [14, 17] cell growth, by enhancing or reducing AKT phosphorylation, respectively.

Consistent with its role as an extracellular matrix protein, EFEMP1's function in cancer was demonstrated to be an effect in the extracellular compartment, by using a purified human recombinant EFEMP1 protein [9, 17, 24]. These observations suggested that EFEMP1 might have potential benefits in cancer therapy, because its effects appear not to be dependent upon intracellular incorporation, and because the kinetics of extracellular incorporation of a therapeutic agent are far more consistent and predictable than those of intracellular incorporation.

Given the potentially conflicting roles of EFEMP1 for cancer biology in general, and the importance of the EGFR signaling pathway in glioma biology, we studied its suppression of EGFR signaling pathways and tumorigenicity in different glioma cells with or without expression of EGFR, and the prognostic values in different sets of glioma patients, dichotomized based on the expression level of *EGFR*. Overall data from glioma experimental models and the patient prognosis values consistently demonstrates that EFEMP1 is tumor-suppressive in gliomas driven by activation of EGFR signaling pathways.

## RESULTS

### EFEMP1 maintains low levels of EGFR signaling activity and tumorigenesis

**Figure 1:**
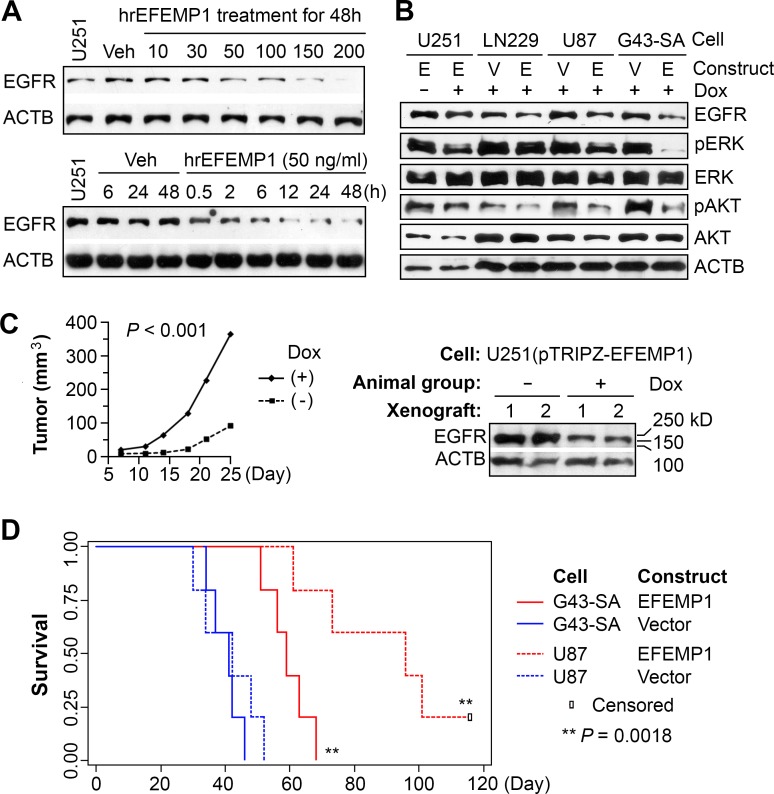
EFEMP1 targets EGFR/EKR/AKT oncogenic pathways to suppress tumorigenicity (A) Immunoblotting of EGFR in U251 cells treated by human recombinant (hr) EFEMP1 at various concentrations (ng/ml) and for various times (h). (B) Immunoblotting of EGFR and downstream targets in glioma cell lines (U251, LN229, U87) and primary culture (G43-SA) transduced by lentiviral vectors pTRIPZ-Empty (Vector) or pTRIPZ-EFEMP1, with or without a 3-day induction of transgene expression by doxycyclin (Dox, 1 ug/ml). (C) Growth curves of subcutaneous (s.c.) xenografts of U251(pTRIPZ-EFEMP1) by measured tumor volumes and western blotting of EGFR in s.c. xenografts with or without providing Dox in water fed to nude mice. (D) Kaplan-Meier survival curves of nude mice with i.c. implantation of EFEMP1- or Vector-transduced U87 and G43-SA (100,000 cells/implantation).

**Figure 2:**
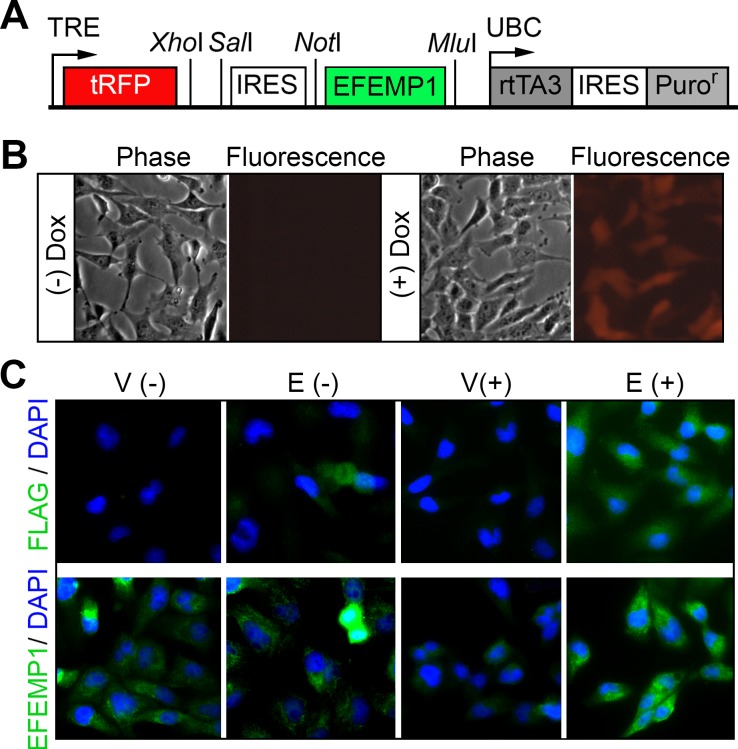
Inducible lentiviral vector overexpressing EFEMP1 (A) Depiction of the pTRIPZ-EFEMP1 construct that co-expresses turboRFP and EFEMP1 (with or without an *N*-terminal FLAG) via the TRE promoter induced by doxycycline (Dox). (*upper*). (B) Infected glioma cells photographed using an inverted phase-contrast, fluorescence microscope. (C) Immunofluorescence (IF) from antibodies for FLAG and EFEMP1 for ectopic EFEMP1 carrying an *N*-terminal FLAG tag and endogenous/ectopic EFEMP1.

In a prior study, we showed that human recombinant (hr) EFEMP1 protein reduced the EGFR level and AKT phosphorylation (pAKT) in 48-hr treated glioma cell lines [17]. Here show a dose- and time-dependent reduction of EGFR by hrEFEMP1 in one of the studied glioma cell lines, U251 (Figure 1A). Due to batch-to-batch variations of hrEFEMP1 production, which hindered the study of signaling pathways affected by EFEMP1, we made a lentiviral vector that expresses ectopic EFEMP1 (with or without an N-terminal FLAG tag), and infected multiple human high-grade-glioma-derived cell lines (U251, U87, LN229), and a glioblastoma-derived primary culture (G43-SA). Immunoblots showed a high level of EGFR in these glioma cells. The lentiviral construct co-expresses red fluorescent protein (RFP) and EFEMP1 after exposure to doxycycline (Dox), shown by immunofluorescence with antibodies against FLAG and/or EFEMP1 (Figure 2). An increase of overall level of *EFEMP1* mRNA following 24-72 hours induction with Dox was also verified by real-time qRT-PCR.

As shown in Figure 1B, a 3-day induction of ectopic EFEMP1 (without FLAG tag) caused marked reductions of EGFR in LN229, U87, and G43-SA, and a minor reduction of EGFR in U251, compared to the controls, which were either empty-vector-infected cells or cells without Dox induction (verified by fluorescent microscope to lack RFP). A marked reduction of membrane EGFR in U251 was found, and is shown in the following report, which is consistent with reduction of EGFR signaling targets pERK and pAKT, as shown in other cell lines.

In contrast to a minor reduction of EGFR by a transient EFEMP1 overexpression in U251 cells *in vitro*, we found significantly suppressed subcutaneous (s.c.) tumorigenesis from a stable induction of ectopic EFEMP1 in U251 and correspondingly, marked reductions of EGFR, in these s.c. xenografts (Figure 1C). We have previously shown tumor suppression from stable transfection of EFEMP1 that nearly abolished U251's tumorigenicity in both s.c. and intracranial (i.c.) xenograft model systems [17]. Here we showed a similar tumor suppressive effect from Dox-induced overexpression of EFEMP1 in glioma cell line U87 and primary culture G43-SA, based on a comparison with the empty-vector control (Figure 1D).

### EFEMP1 interacts with EGFR, blocking EGF from binding and stimulating EGFR signaling

**Figure 3:**
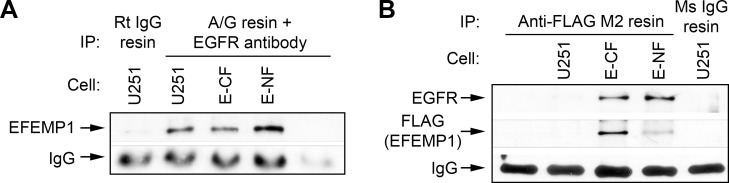
EFEMP1 and EGFR bind *in vivo* Co-immunoprecipitation (IP) of EFEMP1 and EGFR in parental or EFEMP1-transfected U251 (E-CF and E-NF refers to FLAG tags at the *C*- and *N*-terminal ends, respectively), with rabbit anti-EGFR antibody and protein A/G beads (A), or mouse anti FLAG M2 affinity gel (B). Rabbit and mouse IgG agarose resin were used as negative controls.

Given the modular structure of EFEMP1, which has five EGF-like modules and one EGF-like module with insertion [8], we examined interactions between EGFR and endogenous EFEMP1 in U251 and exogenous EFEMP1 in two stable U251 transfectants, with EFEMP1 carrying FLAG tags at either the *C*- or *N*-terminus (E-CF and E-NF, respectively) (refer to [17] for detailed descriptions). In whole-cell lysates of both EFEMP1-transfectants, EFEMP1 was specifically co-immunoprecipitated by antibody against EGFR (Figure 3A), and EGFR specifically by antibody against the FLAG tag (Figure 3B). After the FLAG pull-downs from the whole-cell lysates, FLAG antibody detected a much weaker signaling in E-NF, as compared to that of E-CF. This suggests removal of the FLAG tag along with the signaling peptide during or after the export of E-NF to the extracellular compartment. The similar strong band of EGFR after the FLAG pull-down in both E-CF and E-NF suggests binding of EFEMP1 to EGFR during or after export to the extracellular compartment.

**Figure 4:**
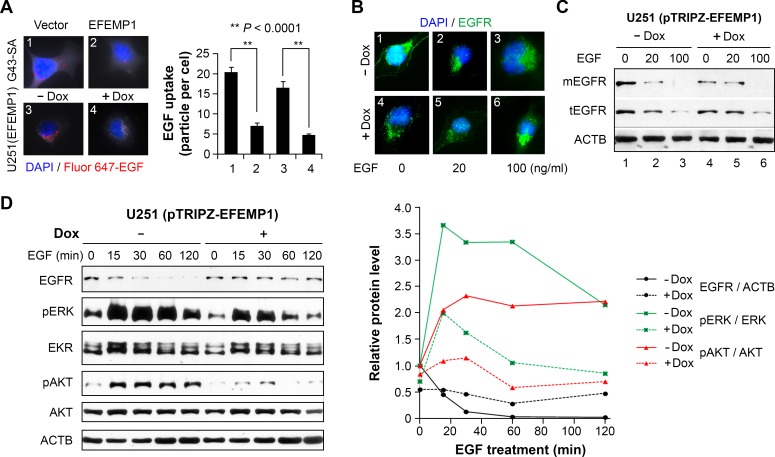
EFEMP1 blocks EGF in activation of EGFR (A) EGF uptake assay in G43-SA and U251 after a 24-hour treatment with Alexa Fluor 647-EGF. (B) Immunofluorescence of EGFR in U251 after a 24-hour treatment with EGF. (C) Immunoblotting of EGFR in U251 whole cell lysate (tEGFR) and membrane-enriched fraction (mEGFR). (D) Immunoblots (left) and plot of normalized densitometry (right) to show the effect of EFEMP1 (a 3-day induction by Dox) on EGFR activation by a low dose of EGF (20 ng/ml). Cells without Dox at each time point were set to unity.

We then examined the effect of transient EFEMP1 overexpression on EGF-mediated activation of the EGFR signaling pathway. First, we examined the competition between EFEMP1 and EGF in binding EGFR, by an EGF-uptake assay, using fluorescently labeled EGF. EGF-uptake is the activation of the canonical EGFR-signaling pathway, and is associated with EGFR internalization [25]. As shown in Figure 4A, EFEMP1 suppressed EGF internalization in both of the glioma cell lines (U251 and G43-SA) examined. Consistently, EFEMP1 reduced EGFR internalization in the presence of EGF, as shown by immunofluorescence detection of EGFR in low-dose (20 ng/ml) EGF-treated U251 infected with lentiviral vector of pTRIPZ-EFEMP1, where ectopic EFEMP1 expression was induced by Dox for 3 days (Figure 4B). Negative controls were infection of empty vector or no Dox treatment.

In the presence of EGF at a low dose (20 ng/ml), immunoblots of EGFR showed reduction of membrane-bound EGFR (mEGFR) in no-Dox control cells, but were unchanged in Dox-treated cells (Figure 4C, *Lanes* 1, 2, 4, and 5). A high-dose (100 ng/ml) EGF treatment diminished the effect of EFEMP1 (Figure 4C, *Lanes* 3 and 6). Overall, the data suggest that EFEMP1, like EGF, binds to EGFR in the extracellular compartment. In contrast to EGF, EFEMP1 inhibits EGFR internalization, but having a long-lasting effect to maintain a low membrane bound EGFR..

We then examined the ability of EFEMP1 to block EGF-mediated activation of EGFR signaling pathways by immunoblotting whole cell lysates of EFEMP1-infected U251 cells. In control cells cultured without Dox, there was canonical EGFR signal activation by EGF, with time-dependent reduction of EGFR and a rapid increase and maintenance of high phosphorylation levels for ERK and AKT (solid lines in Figure 4D). In contrast, these striking changes were nearly abolished by expression of ectopic EFEMP1 induced by a 3-day exposure to Dox (dashed lines in Figure 4D).

### Differential expression of EFEMP1 in gliomas is not correlated with EGFR expression, but does affect patient prognosis

**Figure 5:**
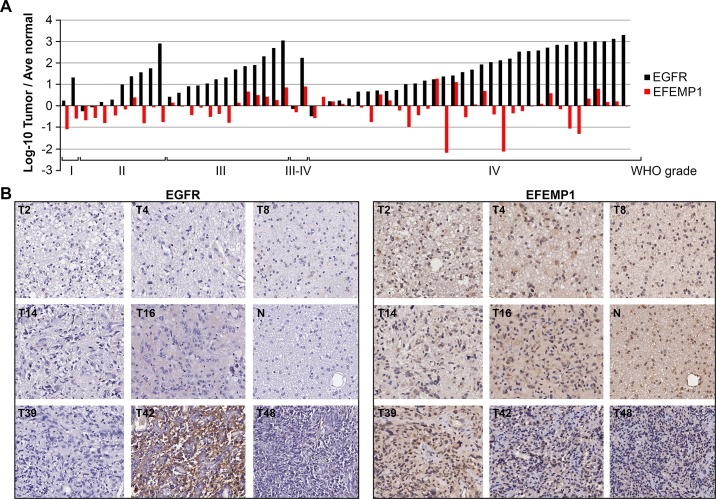
Astrocytic glioma tissue arrays for EGFR and EFEMP1 (A) Relative immunohistochemical intensity of EFEMP1 (red) and EGFR (Black) in 62 gliomas of various grades, according to the World Health Organization (WHO) grading system, normalized to averages of three normal cortical tissues. (B) Representative images of consecutive tissue sections from grade I pilocytic astrocytoma (T2), grade II astrocytoma (T4) and diffuse astrocytoma (T8), grade III anaplastic astrocytoma (T14, T16), normal cortex (N), and grade IV glioblastoma multiforme (T39, T42, T48).

The oncogenic effect of EGFR overexpression in tumors has been well documented; it is commonly observed in malignant gliomas and, in large part, results from *EGFR* gene amplifications [26]. However, *EGFR* expression levels have no prognostic value regarding overall survival of glioma patients [27]. To determine if there is a correlation between protein expression levels of EFEMP1 and EGFR in gliomas, we tested a tissue microarray (TMA) of astrocytic gliomas (N = 65) with antibodies for EGFR and EFEMP1. Comparing to the average of three normal cortex tissues, EGFR was more than 2-fold over-expressed in 82% of tumors and more than 10-fold overexpressed in 77% of tumors. In contrast, EFEMP1 was under-expressed (less than half of the average of the normal cortex tissues) in 40% and overexpressed (more than 2-fold of the normal cortex tissues) in 19% of all tumors, without any significant correlation with tumor grade (Figure 5). Spearman correlation coefficients showed no significant correlation for EFEMP1 and EGFR (Log10(T/N) intensity), with R = 0.27 [95% CI = 0.02 - 0.48; *P* = 0.038]. The significance level was set at *P*<0.01 in order to adjust for the multiple comparisons without overinflating Type II error.

In a published multivariate prognostic model for gliomas, based on quantitative gene expression data, we have shown that the *EGFR* expression level lacked prognostic value in an astrocytic glioma set comprised of 100 glioblastoma multiformes (GBMs) and 49 anaplastic astrocytomas (AAs), but had a favorable effect on overall survival (OS) of patients with oligodendroglial tumors (OTs, n=45) [28]. In 166 glioma cDNA samples from that study, (95 GBMs, 36 AAs, and 35 OTs), we quantified *EFEMP1* and obtained the absolute ratio of *EFEMP1* to *ACTB*, using same-standard-based, real-time qRT-PCR. Spearman rank correlation coefficient on gene expression data of EFEMP1 and EGFR (log10 transformation after a 0.1 offset (log10(x+0.1)) was consistent with protein data shown above; with R=0.16 with [95% CI = 0.01 - 0.31; *P* = 0.036].

Cox proportional hazards regression analysis was then carried out using both log-scaled ratios of *EFEMP1* and *EGFR* vs *ACTB* (continuous variables) and dichotomized variables for these 166 gliomas. When *EFEMP1* expression was treated as a continuous variable, a negative value (-0.182) of the log hazard ratio (HR), and a *P* value = 0.097 in a likelihood ratio test, together suggests a favorable prognostic value for GBM, but lacking prognostic values for AA and OT. We then dichotomized *EFEMP1* and *EGFR* expression variables As shown in Table 1 (left columns), the expression level of *EGFR* lacked a prognostic value, whereas the expression level of *EFEMP1* correlated significantly with a favorable patient prognosis (*P* = 0.037) in overall gliomas. The favorable prognostic value of *EFEMP1* was shown also in GBM (HR) = 0.7 and a 95% confidence interval = (0.4 - 1.1), but unfavorable prognostic value of EFEMP1 was implicated in OT, with HR=2.4 and a 95% confidence interval = (0.5 - 11) (Table 1).

**Table 1:** Prognostic effect of EGFR and EFEMP1 in malignant gliomas based dichotomized subtype

EGFR/ACTB (cutoff 0.05)		EFEMP1/ACTB (cutoff 0.06)				
All gliomas^a^		All gliomas		GBM (95 pts, 88 died)	AA (36 pts, 23 died)	OT (35 pts, 18 died)
1.0 (0.7, 1.4)	P = 0.85	0.6 (0.4, 1.0)	P = 0.037	0.7 (0.4, 1.1)	1.0 (0.4, 2.3)	2.4 (0.5, 11)

a Hazard ratio (HR) adjusted by histology, age and recurrent status. The variation is shown with the 95% confidence interval and P value from a likelihood ratio test.

**Figure 6:**
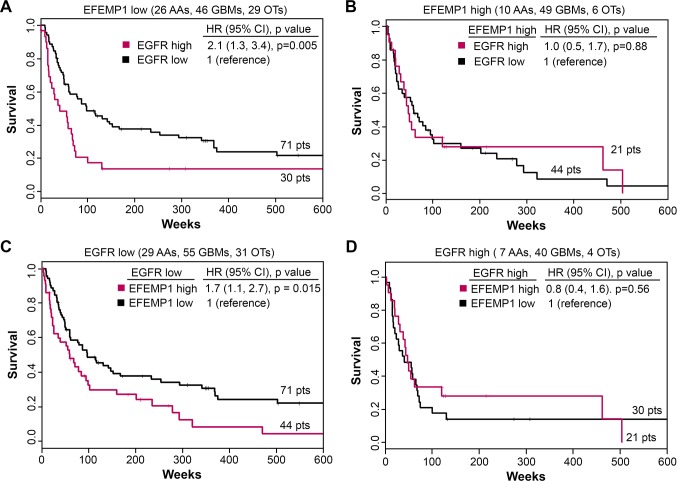
Interdependent prognostic value of *EFEMP1* and *EGFR* expression levels for gliomas (A-B) Kaplan-Meier survival curves for two groups of glioma patients dichotomized based on *EFEMP1* (cut point 0.06 was for *EFEMP1*/*ACTB*). Red denotes *EGFR*-high tumors. Black denotes *EGFR*-low tumors. (C-D) Kaplan-Meier survival curves for two groups of glioma patients dichotomized based on *EGFR* (cut point 0.05 was for *EGFR*/*ACTB*). Red denotes *EFEMP1*-high tumors. Black denotes *EFEMP1*-low tumors.

We then analyzed the prognostic value of *EGFR* in gliomas dichotomized according to high versus low expression level of *EFEMP1* and also the prognostic value of *EFEMP1* in the same set of gliomas dichotomized according to high versus low expression value of *EGFR*. A log-rank test showed a significant difference among the four groups of gliomas (*P* value = 0.010). In the *EFEMP1*-low glioma subset (N = 101), *EGFR* showed a significant, unfavorable, patient prognosis, with HR = 2.1 and a 95% confidence interval = (1.3 – 3.4). In contrast, in the *EFEMP1*-high glioma subset (N = 65), *EGFR* data analysis had no prognostic value (Figure 6A-B). In the *EGFR*-low glioma subset (N = 115), *EFEMP1* data analysis showed a significant, unfavorable, patient prognosis, with HR = 1.7 and a 95% confidence interval = (1.1 – 2.7), while in the *EGFR*-high glioma subset (N = 51), the *EFEMP1* data lacked any significant prognostic value (Figure 6C-D).

## DISCUSSION

The experimental data described above demonstrated the tumor suppressive function of EFEMP1, which operates by attenuating and blocking EGF-stimulated EGFR signaling activities, and is consistent with the patient outcomes predicted based on the EGFR level in the subset of gliomas expressing a low level of EFEMP1 (Figure 6A). This study extends our prior findings that EFEMP1 exerts an overall tumor suppressive effect in GBM, is an important prognostic variable correlating with patient survival time, and suppresses tumorigenicity of human glioma cells with high expression level of EGFR in both i.c. and s.c. xenograft models [17]. This effect was reproduced in i.c. xenograft models with another human malignant glioma line, U87, and with a GBM primary culture, G43-SA (Figure 1).

The mechanisms underlying EFEMP1's tumor suppressive function in glioma has been shown to be mediated through EFEMP1's binding to EGFR (Figure 3). Interaction between EFEMP1 and EGFR has also been reported in pancreatic carcinoma cells [24]. However, the consequence of EFEMP1-EGFR binding appears to be organ-specific. In gliomas, our data showed that EFEMP1 binding to EGFR blocked EGF-mediated activation of PI3K and MAPK signaling pathways (Figures 4), while in pancreatic carcinoma cells it was shown to promote these activities [24].

In this study, data from an astrocytic glioma tissue array showed high expression of EFEMP1 in cortical tissues, and de-regulated expression in tumors, with EFEMP1 under-expressed in 40% of the tissues, over-expressed in 19% of them and unchanged in the remaining tissues, as compared to the expression level in cortical tissues (Figure 5). These results are different from the finding that Fibulin-3 (EFEMP1) was uniquely up-regulated in malignant gliomas, a conclusion based on microarray data [22]. In the adjacent sections in tissue array analysis, EGFR was demonstrated to have low expression in cortical tissues and to be up-regulated in the majority (82%) of tumors; this is consistent with findings of EGFR amplification and overexpression in gliomas that are well documented in the literature and in a recent report from The Cancer Genome Atlas Network (TCGA) [26].

Our prognosis study of glioma patients classified according to *EFEMP1* expression level, demonstrated, for the first time, a significant unfavorable value for *EGFR* (Figure 6A). However, for gliomas, *EGFR*'s prognostic value can only be properly interpreted in the context of *EFEMP1*. High *EGFR* expression is functionally mitigated by high *EFEMP1* expression, whereas low *EFEMP1* expression identifies the subset of glioma patients for whom anti-EGFR therapies might be the most beneficial. Our finding that *EFEMP1* levels in the low-*EGFR subset* correlate positively with poor patient outcome (Figure 6C). It suggests an oncogenic role of EFEMP1 in gliomas that do not have high EGFR expression, or where tumor growth was not largely driven by EGFR-activated signaling. It has been shown in glioma stem-like cells EFEMP1 activates/depends NOTCH signaling to promote cell invasiveness [23, 29]. By combining findings of poor patient prognosis for gliomas with heightened *EGFR* levels but low *EFEMP1* expression, and by clarifying the EGFR-dependent nature of tumor suppression by EFEMP1, our data have provided a rational for low expression of *EFEMP1* being a predictive factor for selecting patients who would benefit from anti-EGFR therapy. Anti-EGFR drug is currently being tested in clinical trials using cancer patients [30].

## MATERIALS AND METHODS

### Ethics Statement

Frozen and fresh glioma specimens were provided by the Tissue Banks of University of California, Irvine and Winthrop P. Rockefeller Cancer Institute Bio Repository at UAMS, with Institutional Review Board approval.

### Animal work on subcutaneous (s.c.) and intracranial (i.c.) xenografts and analysis

The animal work was approved by the Animal Care and Use Committee (IACUC) of the University of California, Irvine. For studies using intracranial (i.c.) xenografts, glioma cells (1 × 10^5^ / 3 μl DMEM/F12) were injected into the frontal lobe of 4-6 week old, female, nude mice (strain NCrNu-M, Taconic, Hudson, NY), following IACUC approved surgical procedures. After i.c. implantation, mice were observed daily and periodically weighed for moribund signs (hunchback posture, marked weight loss and gait impairment). Mice were euthanized when they developed brain-damage symptoms (ataxia, hemiparesia, etc) and/or 20% body weight loss, and the following day was recorded as the survival date for survival analysis.

For studies using subcutaneous (s.c.) xenografts, cells (1 × 10^6^ cells / 50 μl DMEM/F12) were subcutaneously injected into nude mice, anterior to their right and left thighs, on both sides. Tumor measurements were taken every 3-4 days after implantation, and tumor volume was calculated using the formula V = (L*W^2^)/2 (L, length; W, width). Mice were euthanized at a predetermined time of the experiment or when tumor volume exceeded 1.5 cm^3^.

Induction of EFEMP1 expression was achieved by providing mice with water containing 1 mg/mL Dox throughout the experiment. Overall survival of mice bearing intracranial glioma xenografts was estimated using Kaplan-Meier survival curves, and the *P* values were from Log-Rank statistics on pair-wise comparisons of the two groups using Cox Regression. SAS versions 9.2 and 9.3 (The SAS Institute, Cary, NC) were used for all analyses.

### Prognosis analysis of EGFR and EFEMP1 expressions in gliomas

The cDNA samples of gliomas and quantification of *EGFR* and *EFEMP1* have been described in our prior studies [17, 27]. Briefly, we used AqRT-PCR technology [31], with a single standard containing marker and reference genes and gene-specific PCR primers for *EFEMP1* and *ACTB* (Ziren Research LLC, Irvine), to obtain their absolute expression ratios in cDNA samples. In this study, the gene expression data were from 167 glioma cDNA samples. One patient was missing follow-up data, so analyses were based on 166 patients. The completeness of overall survival (OS) data was shown in our previous study [8]; for glioblastoma multiforme (GBM, WHO grade IV, 92% deaths, not including those for whom contact had been lost for over 5 years), almost mature for anaplastic astrocytoma (AA, WHI grade III, with 67% deaths), and half mature for oligodendroglial tumors (OT, 49% deaths).

Cox proportional hazards regression analysis was done on dichotomized gene expression variables. The cut-offs were chosen so that the group that was high for both markers had at least 20 patients. This was done by inspecting the joint distributions of the two markers. Hazard ratio (HR) values and 95% confidence intervals were shown for gene expression variables to overall survival of patients.

### Glioma tissue microarray (TMA)

Astrocytic glioma TMAs were fabricated by the Shanghai Outdo Biotech Co., Ltd. The arrays contained 65 individual tissues including pilocytic astrocytoma, astrocytoma, diffuse astrocytoma, anaplastic astrocytoma, normal cortex, and glioblastoma multiforme. IHC was performed on arrayed tissue samples using antibodies of EGFR (Maixin-Bio, MAB0196) and EFEMP1 (Abgent, AP9095a), following standard IHC procedures. Briefly, after deparaffinization and rehydration using a Leica autostainer XL ST5010 system, the TMA slides were pretreated with 10mM sodium citrate buffer (pH 6.0) for 5-10 minutes in a microwave for antigen retrieval. The endogenous peroxidase was quenched by adding the hydrogen peroxide (3% H_2_O_2_ in 70% methanol) at room temperature for 15 minutes. After washing, the slides were blocked for 30min. The blocking buffer was removed and the slides were then incubated 1 hour with primary antibody at 1:100 dilution at room temperature. Slides were washed with PBS solution and further incubated with DAKO Envision+/HRP for 30 min at room temperature. Detection was done by 3, 3′-diaminobenzidene (DAB kit, DAKO, Denmark), following the manufacture's protocol. Slides were counterstained with hematoxylin before microscopic analysis.

The expression levels of EGFR and EFEMP1 in TMA were measured by analyzing the staining signal intensity using an Aperio image scope v11 (Aperio, USA). Briefly, the built-in positive pixel count v9 algorithm was used to measure the densitometry of the digital image (X 400), and the counted positive pixels were converted to three intensity bins. The relative log ratio was taken from comparison of individual tissues to the mean of three normal cortical tissues.

### Glioma primary cultures and cell lines

The human glioma cell lines (LN229, U251, and U87) were obtained from Dr. Alfred Yung, M.D. Anderson Cancer Center, University of Texas. GBM-derived primary culture line G43-SA were described in [32], with cell line authentication information provided there. LN229, U251, U87, and G43-SA were cultured in Cell Culture/Petri dishes in DMEM/F12 supplemented with 5-10% fetal bovine serum. All cells were cultured in 37oC humidified CO2 (5%) incubators. Apart from LN229, all the other three glioma lines were cultured in serum-free medium for 2 days prior to analyses for proteins in the EGFR signaling pathway, where LN229 remained in culture medium containing serum.

### EFEMP1 lentiviral vector construction and lenti virus production

The protein-coding sequence (CDS) of EFEMP1 cDNA was PCR-amplified by a 5′ primer carrying a Kozak site with or without a FLAG tag at the *N*-terminal of CDS, and 3′ primer carrying a *Xba* I restriction enzyme site, cloned into a TA cloning vector. EFEMP1/pCR2.1 clones were selected for carrying a *Not* I restriction enzyme site upstream of the insertion. After sequencing verification, an error-free EFEMP1-TA clone was subcloned into a modified pBS-KS(+) vector with IRES (from pIRES, Invitrogen) inserted upstream of EFEMP1. Then IRES-EFEMP1 was subcloned into pTRIPZ-Empty (Open Biosystems) via *Xho*I and *Mlu*I. Generation of infectious lentivirus was done by co-infecting HEK-293T with pTRIPZ-Empty (Vector) or pTRIPZ-EFEMP1, along with packaging plasmid psPAX2 and envelope plasmid pCMV-VSVG, as described previously [17]. Two days after co-transfection, culture medium containing lentivirus was filtered (0.45 μm) and applied to glioma cell cultures. The infected glioma cells were selected for 1-2 weeks in culture medium containing 1.25 μg/ml puromycin prior to analysis.

### Co-immunoprecipitation (Co-IP)

Exponentially growing U251 parental and stable transfectants with FLAG-tagged EFEMP1 were harvested by scraping and sonicated in radioimmunoprecipitation assay buffer (RIPA) containing 1X cocktail of protease inhibitor (Roche), then centrifuged at 14,000 rpm for 10 min at 4°C, and applied to Co-IP using unconjugated EGFR antibody with Protein A/G Plus agarose bead (Santa Cruz Biotechnology, Santa Cruz, CA) or anti-FLAG M2 affinity gel (Sigma-Aldrich, St. Louis, MO), following the manufacturer's protocols. Cell lysate (~500 μg protein in 260 μl) was used for each pull-down, with 5μg of EGFR (antibody vs protein lysate =1:100) antibody and 40 μl of A/G resin used for EGFR-pull downs, or 40 μl of anti-FLAG M2 affinity gel for FLAG-pull downs. Negative controls for both pull down assays were rabbit or mouse IgG agarose beads (Cell Signaling) added to the cell lysate of U251, and A/G or anti-FLAG M2 resins added to RIPA buffer. After binding (4oC overnight), the resins was centrifuged at 6000 rpm for 30 sec at 4°C. Supernatant fractions were carefully removed. The resin/ bead was washed three times with the cold RIPA or TBS buffer (50 mM Tris-Cl, 150 mM NaCl, pH 7.4) to remove on-specifically bound proteins then eluted in 2 X SDS sample buffer and boiled for 3 min prior to loading onto SDS-PAGE gels for immunoblotting using antibodies for EFEMP1 (1:500, Aviva Systems), FLAG-M2 (1:1,000, Sigma-Aldrich), and EGFR (1:1000, Cell Signaling)

### EGF-uptake assay

Glioma cells transduced with pTRIPZ-Vector or pTRIPZ-EFEMP1 lentiviral vectors were plated onto ploy-l-lysine (15mg/ml)-coated chamber slides for 24 h in DMEM-F12 medium containing 5% bovine serum. Ectopic EFEMP1 expression was induced by Dox (1ug/ ml) for 2 days. Prior to the EGF uptake assay, cells were washed, and incubated in pre-warmed uptake medium (DMEM containing 15mm HEPES, PH7.2, and 1% BSA) for 1h, then incubated on ice for 30 min in uptake medium containing 2 ug/ml of Alexa Fluor 647-EGF (Invitrogen), washed with ice-cold PBS and then incubated in pre-warmed uptake medium for 15min. Cells were placed on ice and incubated in acidic PBS (pH=4) for 5min to remove the unabsorbed fluorescent EGF, then washed twice with ice-cold PBS and fixed in 4% paraformaldehyde. The intensity of EGF fluorescence was measured by the Image J 1.47v program (NIH, USA), based on images taken by a fluorescent microscope with a 100X lens. First, the image was imported in TIFF format and then the color channels were separated and converted to gray scale images. The edge of a cell was selected as the region of interest using the brush tool. Ten images for each group were analyzed.

### Immunofluorescence and immunoblotting

U251(pTRIPZ-EFEMP1) cells were grown in chamber slides in culture medium without or with doxycycline (1ug/ml) for 2 days, then serum-starved overnight prior to adding EGF to various final concentrations. After a 24 h EGF treatment, cells were subjected to immunofluorescence detection of EGFR. In brief, cells were rinsed with cold PBS, fixed by 4% PFA for 10 min and blocked by 10% donkey serum for 0.5-1 h at room temperature, then incubated with EGFR antibody (1:1000 blocking solution) overnight at 4°C. Alexa 488nm donkey anti-rabbit IgG (Invitrogen) was then applied for 1 h at room temperature, mounted in medium containing DAPI, and then pictures were taken using a fluorescence microscope with a 60X lens.

Similarly treated cells to those described above were subjected to membrane-enrichment by biotin-binding of protein in the cell membrane, followed by streptavidin-pull down, using the EZ-Link Sulfo-NHS-Biotinylation Kit (Pierce). Briefly, cells were washed by cold PBS and biotinylated with 0.1 mg/mL of Sulfo-NHS-LC-Biotin (Pierce) for 30 min at 4°C. Then cells were washed with cold PBS containing 0.1M glycine to quench the unreacted biotin. Cells were lysed in RIPA buffer containing protease inhibitor, and cell debris was removed by centrifugation (8000 g for 10 min at 4°C). Cleared cell lysates were further incubated with streptavidin-conjugated beads (Pierce) for 45min at 4°C, and the precipitated membrane proteins were then subjected to immunoblotting and compared to the whole cell lysate immunoblotting without biotin-streptavidin precipitation.

Antibodies for EGFR, Akt (AKT), phopho-Akt (Ser473) (pAKT), Erk1/2 (ERK), phopho-Erk1/2 (pERK), and NOTCH1 were from Cell Signaling, and Actin (ACTB) from EMD Bioscience, with dilutions based on the manufacturer's recommendations.
